# Effects of straw return on bacterial communities in a wheat-maize rotation system in the North China Plain

**DOI:** 10.1371/journal.pone.0198087

**Published:** 2018-06-07

**Authors:** Dali Yu, Zhiguo Wen, Xiumei Li, Xiaojun Song, Huijun Wu, Peilong Yang

**Affiliations:** 1 Key Laboratory for Feed Biotechnology of the Ministry of Agriculture, Feed Research Institute, Chinese Academy of Agricultural Sciences, Beijing, P. R. China; 2 School of Life Sciences, Qilu Normal University, Jinan, P. R. China; 3 Institute of Agricultural Resources and Regional Planning, Chinese Academy of Agricultural Sciences, Beijing, P. R. China; Agroecological Institute, CHINA

## Abstract

Straw return plays an important role in reducing the use of chemical fertilizer, promoting soil carbon sequestration, thus maintaining soil fertility and alleviating environmental pollution. To examine the effects of straw return on soil bacterial communities, quantitative PCR and high-throughput sequencing approaches were used to analyze the bacterial abundance and community structures at the depths of 5–25 cm and 25–45 cm in the soils under six-year continuous straw return and removal treatments in Langfang, Hebei, the North China Plain. As a result, straw return had no effects on soil chemical properties, bacterial abundance, richness or diversity at both soil depths. In contrast, vertical distributions of available nitrogen and available potassium were affected. Similarly, straw return also changed the vertical distributions of *Proteobacteria* and *Chloroflexi*. Principal coordinate analysis based on weighted UniFrac distance matrix indicated a moderate separation of the bacterial community in the soil treated with straw return from that with straw removal at 5–25 cm depth, but they were not distinctly distinguished at 25–45 cm depth. T-test identified increased abundance of *Candidatus Latescibacteria* in the soil under straw return treatment at 5–25 cm depth but no differentially abundant phyla at 25–45 cm depth was found. These results suggested a selection effect from the six-year continuous straw return treatment and the soil bacterial communities were moderately changed.

## Introduction

Soil bacteria are important components of agroecosystems and represent the major driving force of soil organic matter and nutrient cycling [1]. Due to rapid response to fertilization, soil bacteria have been selected as an important indicator of soil quality [2]. For example, denitrification potential under different fertilization regimes was closely coupled with the shift in denitrifying bacteria communities resulting from the variation of properties in black soil [3]. After a long time, such shift may lead to alternations of the soil function and quality. Therefore, in order to maintain the soil sustainability, it is important to monitor dynamics of the soil bacterial communities.

Agriculture production in China has largely increased in the past fifty years due to high inputs of chemical fertilizer [4]. However, long-term excessive use of chemical fertilizer caused serious soil degradation and environmental pollution [5, 6]. As one of the most economic and effective farming management practices, straw return is widely used to reduce the use of chemical fertilizer, promote soil carbon sequestration and consequently maintain soil fertility [7, 8].

The effects of straw return on soil bacterial communities have been investigated worldwide. In north-central China, long-term maize straw return (30 years) was shown to increase the abundance of Gram-negative (Gm^-^) bacteria, but had no effects on Gram-positive (Gm^+^) bacteria, total bacterial or actinomycetes biomass in a summer maize–winter wheat cropping system [9]. In another study in central China, straw return significantly affected the bacterial abundance, composition and diversity in a rice–wheat cropping system after two cycles of annual rice-wheat rotation [10]. And in central Mexico, a long-term field experiment demonstrated that straw return influenced bacterial community structure significantly and altered the abundances of *Bacteroidetes*, *Cyanobacteria* and *Gemmatimonadetes* [11]. These studies indicate as an environment-friendly practice, straw return plays a role in changing the soil bacterial communities.

The North China Plain, which has about 18 million hectares of farmland, is the main agricultural production base in China (representing 20% of total Chinese food production) [12]. Double cropping winter wheat and summer maize is the main cropping system practiced in this area [12]. Crop straw was commonly burnt directly in the open field to save time and handling cost before, which led to serious environment problems [13, 14]. Thus burning crop straw in the open field is banned and crop straw is encouraged to be returned to the field currently. However, studies investigating the effects of straw return on soil bacterial communities in this region remain so few. Therefore, a comprehensive study was conducted to examine the effects on soil bacterial composition and diversity of straw return in Langfang, Hebei, the North China Plain.

## Materials and methods

### Experiment site

The experiment site was located at Agricultural Station of Chinese Academy of Agricultural Sciences (39°36’N, 116°360’, and altitude 18 m), in the city of Langfang, Hebei Province, China. This region has a temperate continental monsoon climate and the average annual temperature is 11.9°C. The mean annual precipitation is 550 mm, 80% occurring between June and September. The soil is classified as silt loam according to the FAO soil taxonomy system (World Reference Base for Soil Resources, 2006).

### Experiment design

The field experiment was initiated in 2010 and conducted in a winter wheat-summer maize rotation system, in which winter wheat was directly seeded in October and harvested in June of the next year, and summer maize was directly seeded and grown from June to October. Two treatments, straw removal (CK) and straw return (SR), each with three replicates, were arranged in a randomized block design. Calcium superphosphate (150 kg P_2_O_5_ ha^-1^) and potassium sulphate (75 kg K_2_O ha^-1^) were applied in both CK and SR treatments before tillage. In SR treatment, the straw was crushed into pieces and incorporated into the soil by rotary tillage (about 20 cm depth) after wheat and maize harvest every year. The biomass of wheat (44.7% of total carbon, 0.76% of total nitrogen) and maize straw (38.6% of total carbon, 0.92% of total nitrogen) were about 3000 kg ha^-1^ and 8000 kg ha^-1^, respectively.

### Soil sampling and chemical property characterization

Soil samples were collected in early November, 2016. Five soil cores (3 cm in diameter) were randomly taken from each replicate plot with an area of 67 m^2^. Soil cores were separated into two depths (5–25 cm and 25–45 cm) and mixed by depth to form one composite sample [15]. Each sample was placed in a sterile plastic bag, which was sealed, and transported to the laboratory on ice. The samples were then homogenized, passed through a 2.0 mm sieve and equally divided into two parts. One was air-dried for determination of the chemical properties, and the other was stored at -80°C for further molecular analyses.

Soil pH was measured with a soil to water ratio of 1:2.5 (W/V) using a pH meter [16]. Soil total carbon (TC) and total nitrogen (TN) contents were determined through dry combustion with ground samples (100 mesh) on vario PYRO cube element analyzer (Elementar Corporation, Hanau, Germany). The alkali-hydrolysis and diffusion method was employed to determine the content of available nitrogen (available N) [17]. Soil organic carbon (SOC) content was analyzed through the potassium dichromate oxidation method. Available phosphorus (available P) content was determined by the sodium bicarbonate extraction-colorimetry method described by Olsen et al [18]. And the content of available potassium (available K) was determined using the ammonium acetate extraction-atomic absorption spectrophotometry method [19].

### Soil DNA extraction and quantitative PCR (qPCR) analysis

The total DNA was extracted from soil samples using the MoBio PowerSoil DNA Isolation Kit (MoBio Laboratories, Carlsbad, CA, USA) following the manufacturer's protocol with some modifications. The extracted DNA was checked on 1.0% agarose gel. And the quantity and quality was determined using NanoDrop ND-2000 UV-VIS spectrophotometer (Thermo Fisher Scientific, Waltham, MA, USA).

The qPCR method was used to quantify the copy numbers of 16S rRNA genes with primer set 515F (5ʹ-GTGCCAGCMGCCGCGGTAA-3ʹ) and 806R (5ʹ-GGACTACVSGGGTATCTAAT-3ʹ) [15]. The reaction mixture (20 μL) contained FastFire qPCR PreMix (SYBR Green) (Tiangen, China), 0.3 μM of each primer, 0.4 μL 50×ROX reference Dye, and 1 μL of diluted DNA. The reactions were carried out on an ABI QuantStudio™ 7 Flex Real-Time PCR System (Thermo Fisher Scientific, Waltham, MA, USA) according to the following program: 94°C for 1 min, followed by 40 cycles of 94°C for 15 s, 55°C for 34 s, and 72°C for 15 s [15].To prepare the standard curve, the plasmid DNA was extracted from a clone harboring the correct target insert using a Miniprep kit (Tiangen, China). Then the purified linearized plasmid DNA was serially diluted 10-fold and subjected to qPCR to generate an external standard curve. The qPCR reactions were run in quadruplicate with the DNA from all the samples.

### High-throughput sequencing and bioinformatics analysis

The V4 hypervariable region of the bacterial 16S rRNA gene was amplified using the primer set 515F and 806R tagged with barcode. All PCR reactions were carried out with Phusion®High-Fidelity PCR Master Mix (New England Biolabs, USA) according to the following program: 98°C for 1 min, 30 cycles of 98°C for 10 s, 50°C for 30 s and 72°C for 30s, with a final extension of 72°C for 5 min. After purification and pooled at equal concentrations, the PCR products were sequenced on the HiSeq 2500 platform (Illumina, SanDiego, CA, USA) at Novogene, Beijing, China. All the raw sequencing data were deposited into the National Center for Biotechnology Information (NCBI) Sequence read archive database with accession numbers SRR6337101-SRR6337112.

Briefly, the raw 250 bp paired-end reads were trimmed to remove the barcode and primer sequences and merged using FLASH V1.2.7 [20]. Low-quality splicing sequences were filtered using QIIME V1.7.0, and chimeras were removed through UCHIME algorithm [21, 22]. Operational taxonomic units (OTUs) were clustered at the threshold of 97% similarity by Uparse V7.0.1001 [23]. Representative sequences for each OTU were assigned using the SILVA SSU database, with a minimum supporting threshold of 0.8 [24]. Alpha-diversity indices, including Chao1, Shannon, Simpson, ACE, and Goods_coverage were calculated with QIIME V1.7.0. Rarefaction curves, hierarchically clustered heat map, principal coordinate analysis (PCoA) based on weighted UniFrac distance matrix, and analysis of similarities (ANOSIM) were generated by R software (V2.15.3).

### Statistic analysis

One-way ANOVA was used to analyze the differences of soil chemical properties, 16S rDNA gene abundance, the alpha-diversity indices and the relative abundance of the dominant phyla among different groups. Tukey method at P < 0.05 was used for multiple comparisons of group means. To test the main effects and interactions between straw return and depth, two-way ANOVA was performed. These statistical analyses were conducted using SAS9.2 statistical software (SAS Institute, Cary, NC, USA). Independent samples T-tests were applied to identify differentially abundant bacteria taxa in the straw return vs. the straw removal soils at 5–25 cm and 25–45 cm depths by R software (V2.15.3), at a significance level of P < 0.05.

## Results

### Soil chemical properties and bacterial abundance

The contents of TC, TN, available N, available P, available K, SOC and the soil pH had no significant differences between straw return and removal treated soils at both soil depths after six years (Table 1). But the contents of TC, TN, available P and SOC at 5–25 cm depth were much higher than that at 25–45 cm depth, no matter in straw return or removal treated soils, indicating straw return had no effects on the vertical distributions of these soil nutrients (Table 1). However, the vertical distributions of available N and available K were changed after six-year continuous straw return treatment (Table 1). Additionally, through two-way ANOVA, in contrast with straw return, depth effects were observed in all the soil chemical properties except soil pH, but no significant interactions between straw return treatment and soil depth were detected (Table 1).

**Table 1:** Chemical properties of the soils under different treatments.

Depth (cm)	Treatment	pH	Total C (g kg^-1^)	Total N (g kg^-1^)	Avail N (mg kg^-1^)	Avail P (mg kg^-1^)	Avail K (mg kg^-1^)	SOC (g kg^-1^)
5–25	CK	7.56±0.17a	16.32±0.43a	0.74±0.03a	40.80±5.15ab	28.48±9.06a	113.04±6.12ab	6.29±0.32a
	SR	7.56±0.11a	16.49±0.78a	0.77±0.01a	44.25±6.57a	27.34±3.57a	136.41±26.80a	6.40±0.16a
25–45	CK	7.75±0.01a	13.32±2.01b	0.55±0.04b	28.44±4.97b	9.49±1.17b	96.87±2.06b	4.08±0.36b
	SR	7.65±0.13a	13.37±1.39b	0.58±0.03b	31.22±1.66b	10.49±3.77b	98.22±10.69b	4.60±0.38b
Analysis of variance								
S		ns	ns	ns	ns	ns	ns	ns
D		ns	*	**	**	**	**	**
S×D		ns	ns	ns	ns	ns	ns	ns

Values are means ± standard deviations.

Values within the same column followed by the different letters indicate significant

difference at the level of 0.05 or 0.01.

*Avail N* Available N, *Avail P* Available P, *Avail K* Available K, *S* straw return, *D* depth, *ns* no significant significance.

*P<0.05

**P<0.01.

**Fig 1:**
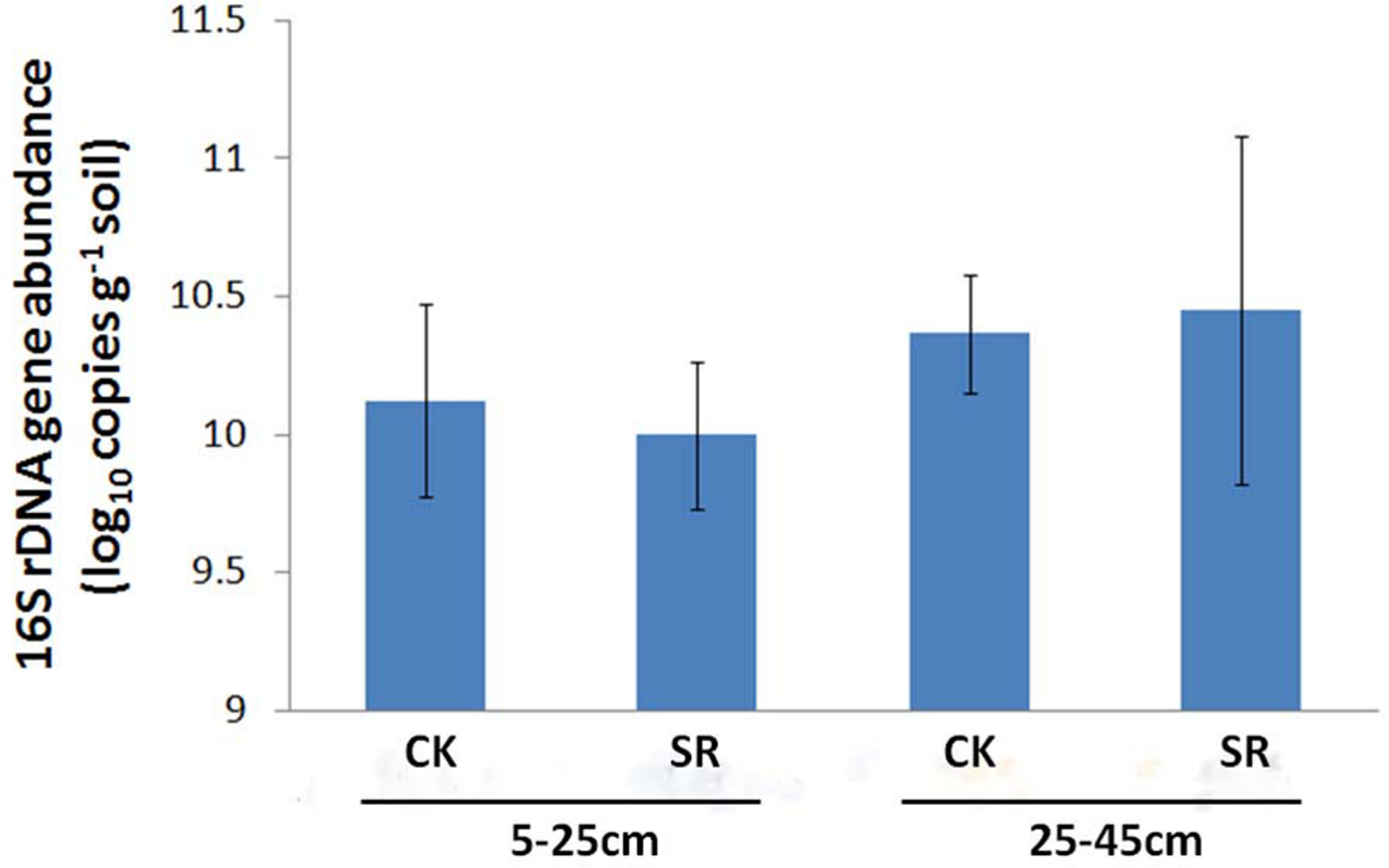
The 16S rDNA gene abundance in soils under different treatments determined using qPCR.

The bacterial abundance was indicated by the copy numbers of 16S rDNA genes. Using qPCR method, the copy numbers of 16S rDNA genes varied from 1.14×10^10^ to 5.80×10^10^ g^-1^ soil, and no significant difference was found between straw return and removal treatments at both soil depths (Fig 1).

### Overview of the sequencing analysis and bacterial alpha-diversity

High-throughput sequencing generated a total of 591,357 paired-end Illumina reads. After sequencing trimming, assembly, quality control and chimera removal, 569,497 high-quality sequences were obtained from 12 soil samples, with an average of 47,458 sequences per sample. And the mean sequence length is 253 bp. Based on subsampling to an equal number of sequences across all the samples (33,239 sequences per sample, according to the minimum sequence number across all samples), clustering of operational taxonomic unites (OTUs) analysis was performed at 97% sequence similarity. As shown in S1 Fig, 6633 unique OTUs were obtained, of which 3378 OTUs were shared among all the four groups, accounting for about two thirds in each group. The rarefaction curves for all samples approached almost plateau phase, suggesting that the sequencing depth was sufficient to capture most bacterial diversity (S2 Fig). It was also confirmed by the Goods_coverage values which were in the range of 0.965–0.985 (S1 Table). High alpha-diversity estimates were observed among all the samples but no significant differences in bacterial richness or diversity as indicated by Shannon, ACE, Chao1 and Simpson indices were detected between straw return and removal treatments (S1 Table). Two-way ANOVA was also performed and no significant depth effect or interactions between straw return and depth were observed (S1 Table).

### Analysis of bacterial community composition

**Fig 2:**
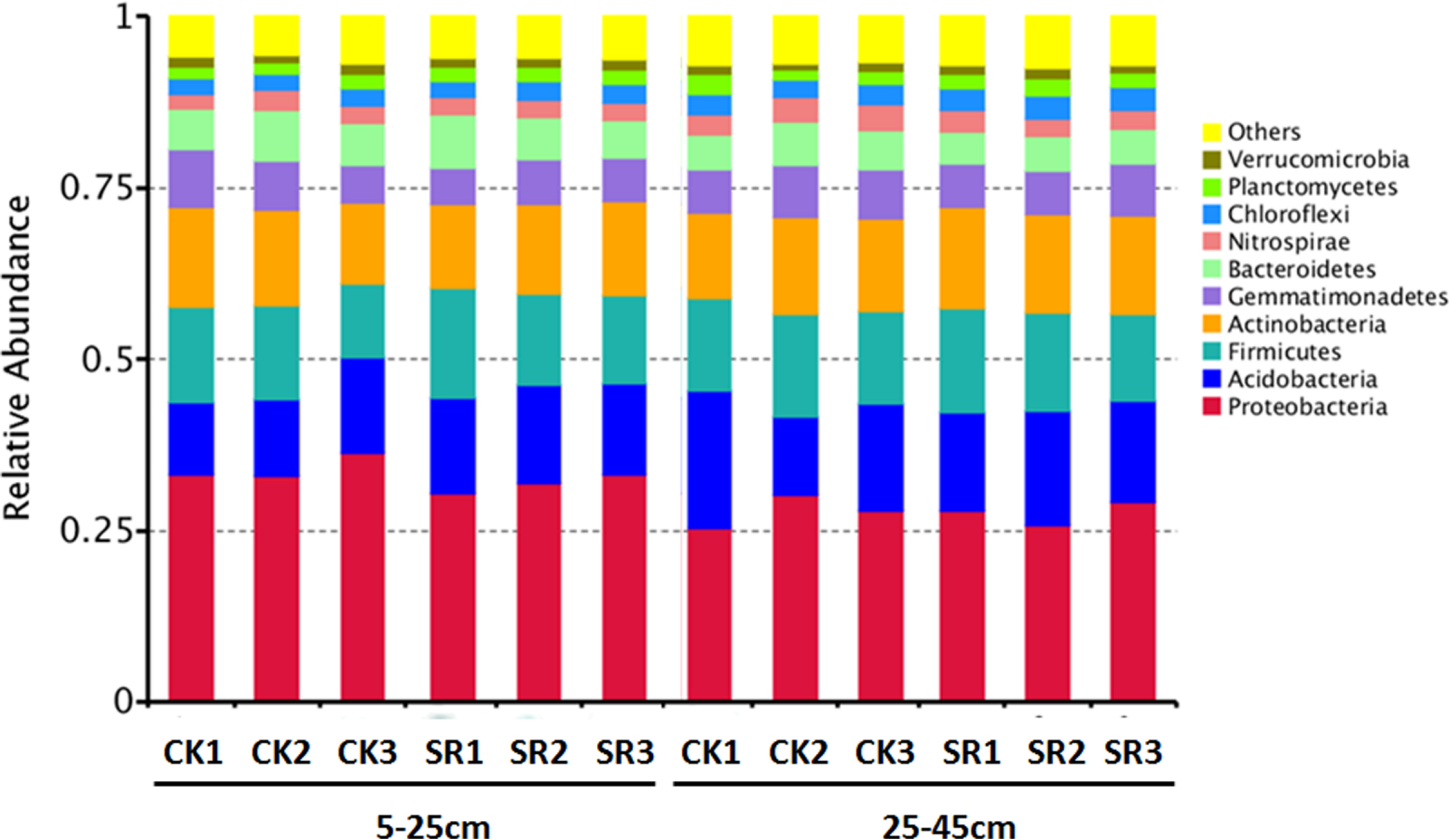
Relative abundance of the ten most dominant bacterial phyla in soil samples under different treatments.

Taxonomic classification of the 16S rRNA gene sequences was performed through annotating each representative OTU tags to the SILVA SSU database using Mothur (V1.33.3) [25]. As a result, 99.11% of the sequences belonged to the domain Bacteria and 0.89% to Archaea. In addition, 39 phyla, 75 classes, 160 orders, 277 families, and 460 genera were identified. Ten most dominant phyla in each sample were shown in Fig 2. *Proteobacteria*was the most abundant phylum, followed by *Acidobacteria*, *Firmicutes*, *Actinobacteria*, *Gemmatimonadetes*, *Bacteroidetes*, *Nitrospirae*, *Chloroflexi*, *Planctomycetes* and *Verrucomicrobia*, accounting for 92.4%-94.4% of all sequences. Though no significant differences in the relative abundance of these ten phyla were observed between straw return and removal treated soils at both depths, variations of the abundance of *Proteobacteria* and *Chloroflexi* between the two soil depths under straw return treatment were different from those under straw removal, showing straw return changed their vertical distributions (S2 Table). It indicated that *Proteobacteria* and *Chloroflexi* may be more sensitive to straw return treatment than other bacteria taxa. Through two-way ANOVA, it was found that straw return increased the relative abundance of *Chloroflexi* and depth had effects on the relative abundance of *Proteobacteria*, *Bacteroidetes*, *Nitrospirae* and *Chloroflexi*, but no significant interactions were observed between straw return and depth (S2 Table).

### Bacterial beta-diversity analysis

**Fig 3:**
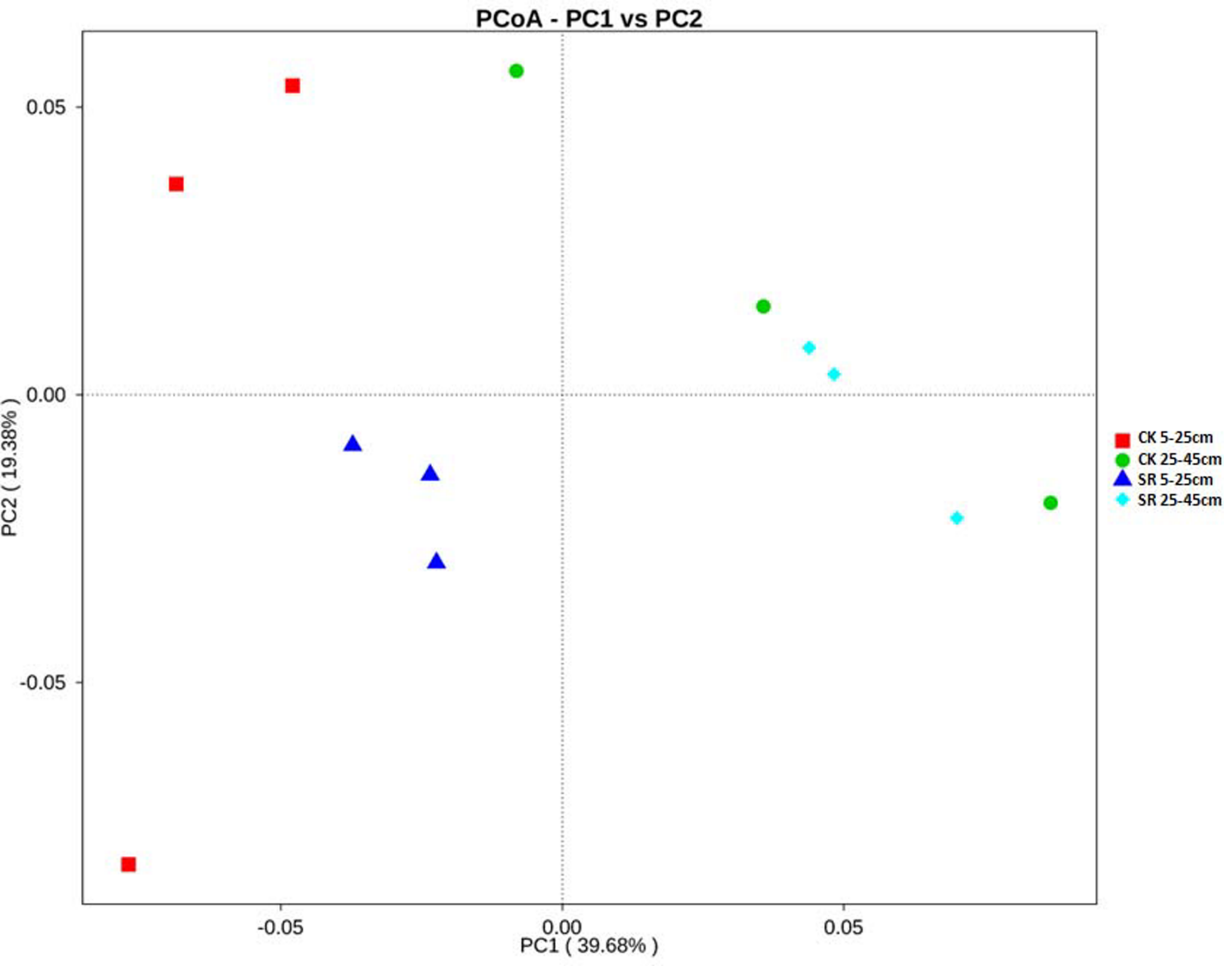
Principal coordinates analysis (PCoA) of the bacterial community compositions in the soils under different treatments based on weighted UniFrac matrix.

**Fig 4:**
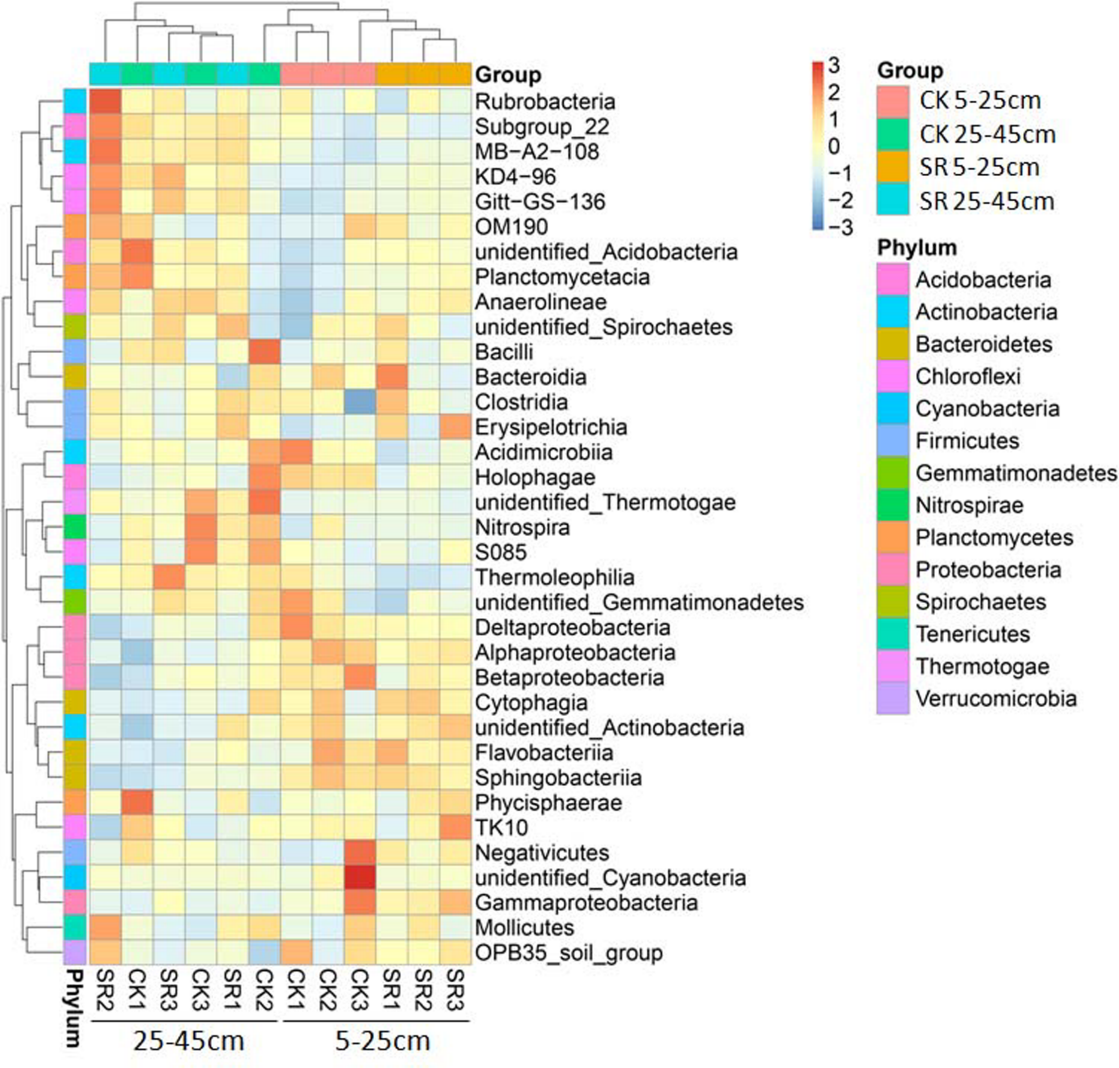
Hierarchically clustered heat map analysis of the dominant bacterial classes in the soil samples under different treatments. The relative percentage of each bacterial classis depicted by varying color intensities according to the legend at the top of the figure. The colour key denotes the Z score indicating correspondence between blue-red colouring and standard deviations from the mean abundance of each bacterial taxon.

PCoA based on weighted UniFrac distance matrix was used to examine the variations of bacterial community compositions. The first and second principal coordinates explained 39.68% (PC1) and 19.38% (PC2) of the total variance, respectively (Fig 3). PC1 separated the 5–25 cm depth from the 25–45 cm depth, although a high variation for the PC2 values was observed. The bacterial communities at 5–25 depth were characterized by negative PC1 values while those at 25–45 cm depth had positive ones. At 5–25 cm depth, samples from straw return treatment formed a unique cluster themselves, separating from the straw removal ones, though a higher degree of variation was observed among the replicates of straw removal. While no distinct discrimination was observed between straw return and straw removal treatments at 25–45 cm depth. These results were consistent with the clear clustering of the dominant bacterial class in the heat map (Fig 4), indicating that the top soil layer was affected more significantly by straw return. However, ANOSIM analysis by R software (vegan package) indicated there were no differences in the bacterial community structures between straw return and straw removal treatments both at the depths of 5–25 cm (R = 0.2593, P = 0.1) and 25–45 cm (R = 0.3333, P = 0.1).

### Differentially abundant bacteria taxa in soils with the straw return vs. the straw removal treatments at 5–25 cm depth

**Fig 5:**
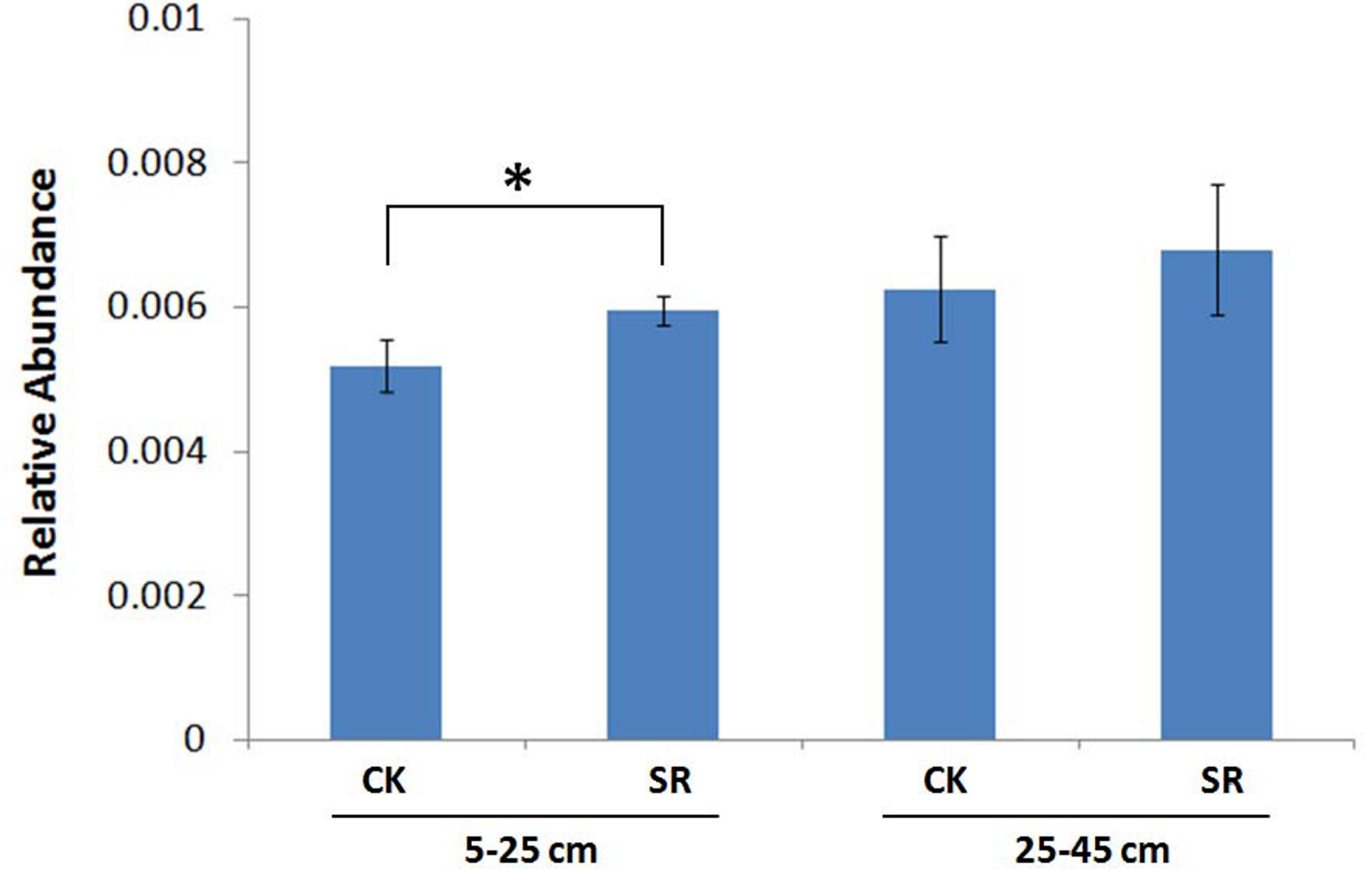
Relative abundance of *Candidatus Latescibacteria* in the soils under different treatments.

In contrast to unweighted UniFrac distance, weighed UniFrac takes abundance of taxa into account and is thus more sensitive to taxa with low abundance. The moderate community separation between straw return and straw removal treatments at 5–25 cm depth indicated that the rare bacterial taxa were affected by the application of straw return to a certain extent. T-test analysis identified the relative abundance of *Candidatus Latescibacteria* increased in the soil under straw return treatment compared with that under straw removal at 5–25 cm depth (Fig 5). However, no differentially abundant phyla at 25–45 cm depth were found.

## Discussion

### Effect of straw return on soil properties

In the present study, a few soil chemical properties were tested. Of them, the soil pH had no significant changes over six years between the straw return and removal treated soils at both soil depths (Table 1). It is consistent with the previous results [16, 26], and means that straw return will not result in soil acidification. In addition, straw return improved the contents of TC, TN, available N, available K and SOC at both depths but the effects were not significant (Table 1). It might be ascribed to the fact that the duration of straw return in this experiment was not long enough. Because these properties are not sensitive to straw return and needs a long period to respond to residue amendments. For example, through field experiments and meta-analysis, it has been found that straw return played important roles in SOC sequestration [8, 27]. However, a short-term (3-year) field experiment conducted in a rice–wheat cropping system at three sites from central to eastern China showed that straw return significantly increased the concentrations of SOC only at two of them [28]. Therefore we infer that straw return can improve the soil fertility but it may take a long time.

### Effect of straw return on bacterial abundance

In this study, bacterial abundance, as indicated by the copy numbers of 16S rDNA genes, was determined using qPCR assay. And no significant differences were found between straw return and removal treatments at both soil depths (Fig 1). A previous phospholipid fatty acid (PLFA) analysis showed that long-term maize straw return (30 years) had no effects on total bacterial abundance in a summer maize-winter wheat cropping system in north-central China even though the contents of SOC was found significantly increased owing to straw return treatment [9]. In contrast, a two-year field experiment showed that straw return significantly increased the bacterial abundance in a rice–wheat cropping system in central China [10]. It has been reported that the bacterial abundance significantly correlated with soil pH and SOC, indicating that the soil pH and SOC could have major effects on it [15, 29, 30]. Furthermore, many other factors can also influence bacterial abundance, including soil texture, climate, fertilization and crop residue quality [31, 32]. These factors altogether may account for the distinct results.

### Effect of straw return on soil bacterial communities

Straw was mainly decomposed by soil microorganisms. It is believed this process generally includes two phases. Bacteria dominate the initial phase while fungi dominate the later one, since bacteria grow faster and are assumed less capable of decomposing recalcitrant compounds than fungi [33, 34]. However, fungal diversity responded as quickly as bacterial diversity indicating that bacteria and fungi may both play important roles during the initial stage [35]. Structures of soil microbial communities also change with straw return treatment. In the present study, high-throughput sequencing approach was adopted to comprehensively investigate the effects of straw return on soil bacterial communities after six years in Langfang, Hebei, the North China Plain.

The richness or diversity of the bacterial communities had no significantly changes after straw return treatment (S1 Table), as reported in the tobacco-rice rotation system [36]. And *Proteobacteria*, *Acidobacteria*, *Firmicutes*, *Actinobacteria*, *Gemmatimonadetes*, *Bacteroidetes*, *Nitrospirae*, *Chloroflexi*, *Planctomycetes* and *Verrucomicrobia* were found to be the dominant bacteria phyla (Fig 2, S2 Table). This is consistent with the previous reports [11, 26]. However, they found more *Bacteroidetes* in the 0–20 cm soil layer under straw return treatment [11, 26]. In our study, no significant differences in the relative abundance of the dominant phyla were observed at both depths. Buts traw return significantly affected the vertical distributions of *Proteobacteria* and *Chloroflexi* (S2 Table). And further two-way ANOVA showed that straw return increased the relative abundance of *Chloroflexi* (S2 Table). The different C:N ratio may account for the differences because nitrogen fertilizer was not used in this experiment.

Though there were no significant differences in the relative abundance of the dominant phyla between straw return and removal treated soils at both depths (S2 Table), T-test identified the increased relative abundance of *Candidatus Latescibacteria* in the soil under straw return treatment at 5–25 cm depth (Fig 5). However, no differentially abundant phyla were found at 25–45 cm depth. It is consistent with the PCoA result and indicated the composition of bacterial community at 5–25 cm depth was moderately affected by straw return treatment while that at 25–45 cm was not (Fig 3).

The presence of *Candidatus Latescibacteria* has been detected across a wide range of terrestrial and marine habits including soils, marine sediments and hydrothermal vents, deep sea hypersaline anoxic lakes, hydrocarbon-impacted environments and wastewater treatment bioreactors [37, 38, 39, 40, 41, 42]. Metabolic reconstruction suggests the prevalence of plant polysaccharide degradation abilities within all “*Candidatus Latescibacteria*” orders and the occurrence of all genes/domains necessary for the production of cellulosome within three orders in datasets recovered from anaerobic locations [43]. These features may make *Candidatus Latescibacteria* sensitive to straw return and could be used as a potential indicator of fertility and quality of soils under straw return treatment.

Moreover, the features considered to responding more quickly to soil management practice changes, such as labile organic carbon fractions, will be checked [28, 44]. And correlations between the chemical parameters and the shift of the bacterial communities will be analyzed to illustrate the mechanism of how straw return affects the shift of bacterial communities.

## Conclusions

In the present study, soil samples treated with six years of continuous straw and removal were collected at two depths (5–25 cm and 25–45 cm) to test for chemical properties and bacterial communities. The results indicated that straw return had no effects on soil chemical properties, bacterial abundance, richness or diversity. However, vertical distributions of available N and available K were affected. Similarly, straw return also changed the vertical distributions of *Proteobacteria* and *Chloroflexi*. In addition, the composition of bacterial community at 5–25 cm depth was moderately affected while that at 25-45cm was not. These results suggested a selection effect from the six-year continuous straw return treatment on the soil chemical properties and bacterial communities. However, we only conducted this study in one site of the North China Plain, and thus further works in more sites with different SOC contents will make it more substantial.

## Supporting information

S1 Fig Venn diagram showing the number of shared and unique OTUs from soils under different treatments. (TIF)

S2 Fig Rarefaction curve of observed species number clustered at 97% sequence similarity for the soil samples under different treatment. (TIF)

S1 Table OTUs, goods_coverage, richness and diversity for bacterial communities from the soils under different treatments. (DOCX)

S2 Table The abundance of dominant phyla from the soils under different treatments. (DOCX)
