# Supplementary figures and images for: Effects of straw return on bacterial communities in a wheat-maize rotation system in the North China Plain
Source: PLoS One. 2018 Jun 7;13(6):e0198087. doi: 10.1371/journal.pone.0198087 (PMC5991650; doi:10.1371/journal.pone.0198087)

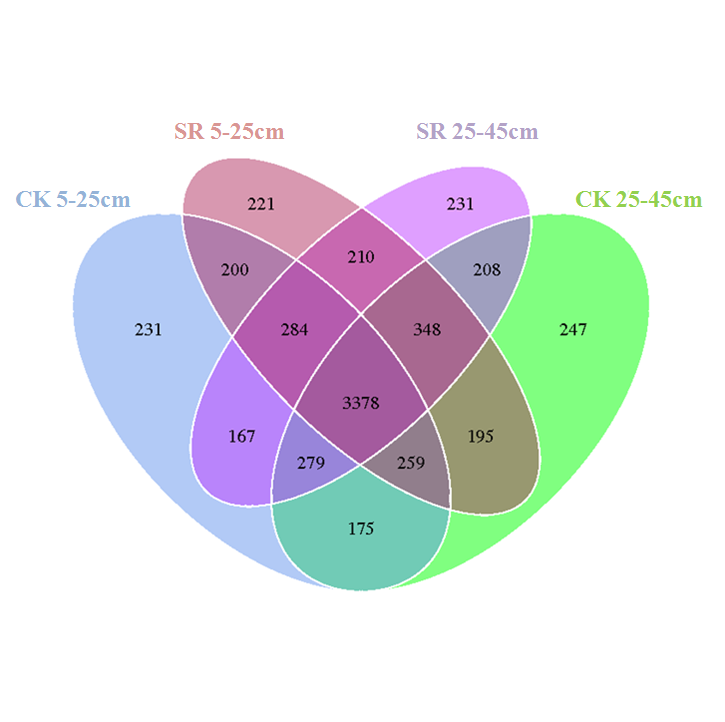

Supplement: S1 Fig — (TIF) [file pone.0198087.s001.tif]

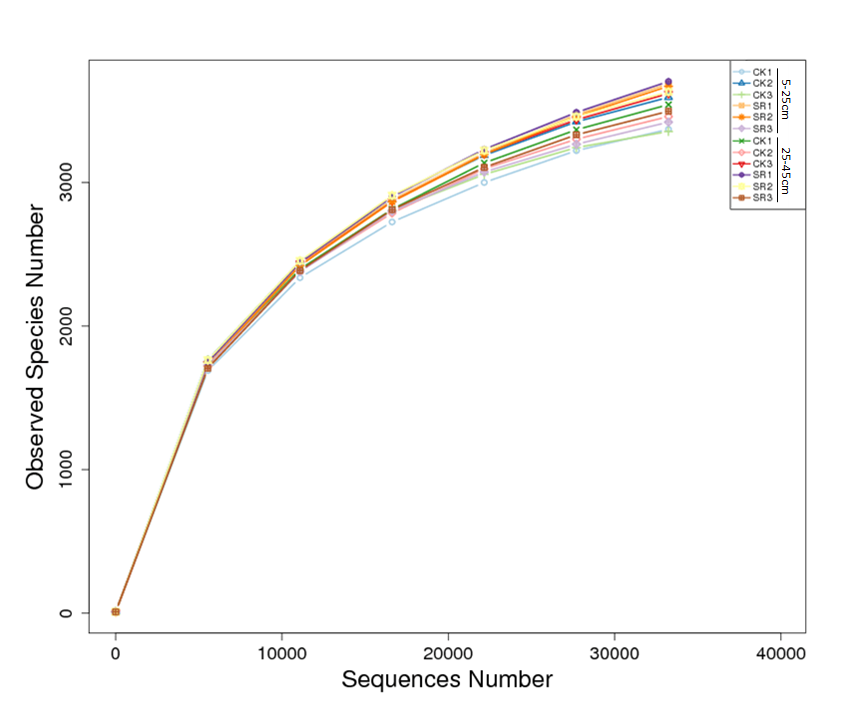

Supplement: S2 Fig — (TIF) [file pone.0198087.s002.tif]
