# Supplementary material for: Effects of straw return on bacterial communities in a wheat-maize rotation system in the North China Plain
Source: PLoS One. 2018 Jun 7;13(6):e0198087. doi: 10.1371/journal.pone.0198087 (PMC5991650; doi:10.1371/journal.pone.0198087)
Supplement: S1 Table — (DOCX) [file pone.0198087.s003.docx]

**S1 Table.** OTUs, goods_coverage, richness and diversity for bacterial communities from the soils under different treatments.

| Depth (cm) | Treatment | OTUs | goods_  coverage | Richness and diversity indices | | | |
| --- | --- | --- | --- | --- | --- | --- | --- |
|  |  |  |  | shannon | simpson | chao1 | ACE |
| 5-25 | CK | 3438.33±133.29a | 0.98±0.01a | 10.23±0.07a | 0.998±0.00a | 3793.41±293.49a | 3975.51±318.37a |
|  | SR | 3591.33±149.49a | 0.97±0.01a | 10.21±0.04a | 0.998±0.00a | 4568.37±549.26a | 4551.09±477.89a |
| 25-45 | CK | 3539.00±80.02a | 0.97±0.00a | 10.21±0.05a | 0.998±0.00a | 4090.15±129.72a | 4257.71±157.01a |
|  | SR | 3609.00±105.09a | 0.97±0.00a | 10.23±0.05a | 0.998±0.00a | 4317.19±419.07a | 4439.89±329.93a |
| Analysis of variance |  |  |  |  |  |  |  |
| S |  | ns | ns | ns | ns | ns | ns |
| D |  | ns | ns | ns | ns | ns | ns |
| S×D |  | ns | ns | ns | ns | ns | ns |

Values are means ± standard deviations. Values within the same column followed by the different letters indicate significant difference at the level of 0.05 or 0.01.

*S* straw return, *D* depth, *ns* no significant significance.

*P<0.05; **P<0.01.
