# Supplementary material for: Effects of straw return on bacterial communities in a wheat-maize rotation system in the North China Plain
Source: PLoS One. 2018 Jun 7;13(6):e0198087. doi: 10.1371/journal.pone.0198087 (PMC5991650; doi:10.1371/journal.pone.0198087)
Supplement: S2 Table — (DOCX) [file pone.0198087.s004.docx]

**S2 Table**. The abundance of dominant phyla from the soils under different treatments.

| Depth (cm) | Treatment | Proteobacteria | Acidobacteria | Firmicutes | Actinobacteria | Gemmatimonadetes | Bacteroidetes | Nitrospirae | Chloroflexi | Planctomycetes | Verrucomicrobia |
| --- | --- | --- | --- | --- | --- | --- | --- | --- | --- | --- | --- |
| 5-25 | CK | 34.10±1.92a | 11.86±1.77a | 12.87±1.81a | 13.46±1.40a | 7.08±1.44a | 6.45±7.96a | 2.57±0.36ab | 2.36±0.15b | 1.81±0.26a | 1.34±0.28a |
|  | SR | 31.89±1.35ab | 13.77±0.47a | 14.08±1.68a | 13.11±0.63a | 6.00±0.65a | 6.46±1.08a | 2.51±0.03b | 2.60±0.16b | 2.13±0.10a | 1.32±0.04a |
| 25-45 | CK | 27.80±2.41b | 15.68±4.25a | 14.03±0.80a | 13.36±0.82a | 7.11±0.63a | 5.65±0.76a | 3.38±0.39a | 2.88±0.32ab | 2.17±0.70a | 1.10±0.17a |
|  | SR | 27.67±1.72b | 15.12±1.26a | 14.13±1.31a | 14.57±0.26a | 6.76±0.76a | 4.90±0.18a | 2.74±0.34ab | 3.32±0.20a | 2.30±0.26a | 1.19±0.19a |
| Analysis of variance |  |  |  |  |  |  |  |  |  |  |  |
| S |  | ns | ns | ns | ns | ns | ns | ns | * | ns | ns |
| D |  | ** | ns | ns | ns | ns | * | * | ** | ns | ns |
| S×D |  | ns | ns | ns | ns | ns | ns | ns | ns | ns | ns |

Values are means ± standard deviations. Values within the same column followed by the different letters indicate significant difference at the level of 0.05 or 0.01.

*S* straw return, *D* depth, *ns* no significant significance.

*P<0.05; **P<0.01.
